# Evidence for a functional adrenomedullin signaling pathway in the mouse retina

**Published:** 2012-05-30

**Authors:** Jan Blom, Thomas J. Giove, Winnie W. Pong, Todd A. Blute, William D. Eldred

**Affiliations:** Boston University, Laboratory of Visual Neurobiology, Department of Biology, Boston, MA

## Abstract

**Purpose:**

Adrenomedullin (ADM) is a small, secreted peptide often associated with vasodilation. However, ADM can also function as a neurotransmitter/neuromodulator, and studies suggest ADM is upregulated in the eye in several ocular diseases. However, no studies to date have described an ADM signaling pathway in the retina.

**Methods:**

PCR, immunocytochemistry, nitric oxide imaging, western blots, and a nitrite assay were used to determine the localization of the components of the ADM signaling pathway in the mouse retina.

**Results:**

We used reverse-transcriptase polymerase chain reaction to show that ADM and its primary receptor, calcitonin-receptor-like receptor, along with its associated receptor activity modifying proteins 2 and 3 are expressed in the retina. Using immunocytochemistry, we detected ADM staining throughout the retina in the photoreceptor outer segments, the outer nuclear layer, Müller and amacrine cell somata in the inner nuclear layer, and some somata in the ganglion cell layer. We found that calcitonin-receptor-like receptor and receptor activity modifying protein 2 had localization patterns similar to ADM, especially in somata in the inner nuclear and ganglion cell layers. Finally, we showed that the ADM receptor was functional in the retina. Stimulation of isolated retinas with ADM increased cyclic adenosine monophosphate– and cyclic guanosine monophosphate–like immunoreactivity, as well as nitric oxide production.

**Conclusions:**

These results are the first to show that ADM and functional ADM receptors are present in the retina. Since ADM is increased in eyes with ocular pathologies such as diabetic retinopathy, glaucoma, retinitis pigmentosa, and uveitis, the ADM signaling pathway may provide a new target for ameliorating these retinal pathologies.

## Introduction

Adrenomedullin (ADM) is a small, secreted signaling peptide first identified in a pheochromocytoma of the human adrenal gland and belongs to the calcitonin gene–related peptide family [1]. ADM circulates in the human plasma at levels of 2–3 pM and is expressed in numerous organs, including cardiac muscle and the kidney [1-3]. ADM is also found throughout the central nervous system (CNS), including the cerebellum and cerebral cortex [4]. ADM may be involved in several ocular pathologies. For example, there is increased ADM in the vitreous humor of patients with diabetes [5,6], in patients with primary open-angle glaucoma [7], and in the aqueous humor of patients with uveitis and vitreoretinal disorders [8], and patients with retinitis pigmentosa have increased plasma ADM [9].

While the earliest discovered activity of ADM was as a vasodilator [1], ADM also functions as a neurotransmitter/neuromodulator, in that the peptide can directly alter the electrical responses of neurons in culture [10] and can elicit calcium transients and activate nitric oxide (NO) production in cultured cortical neurons [11]. In the eye, ADM was originally found in retinal pigment epithelial (RPE) cells [8,12]. Two previous studies have examined ADM in the retina, but they provide little anatomic detail on the localization of ADM, and they do not characterize its downstream signaling or receptors. Zhu et al. [13] performed immunocytochemistry using an ADM antiserum and reported scattered cells in parts of the ganglion cell layer (GCL), the inner and outer nuclear layers (INL and ONL), and the retinal pigment epithelium. Thiersch et al. [14] found upregulation of the ADM gene in response to hypoxic preconditioning.

ADM primarily activates the type III G-protein coupled receptor (GPCR) calcitonin receptor-like receptor (CRLR), when CRLR is coupled with either the receptor activity modifying protein 2 or 3 (RAMP2 or RAMP3) [15,16]. Binding of ADM to its receptor can lead to an elevation of cyclic adenosine monophosphate (cAMP) and activation of protein kinase A (PKA) [1,17]. Xu and Krukoff [11] showed that this activation of PKA can lead to an increase in intracellular calcium, which is consistent with the work of Kuwasako et al. [18], who also showed intracellular calcium mobilization via cAMP after ADM binding to CRLR/RAMP. Other GPCRs have some affinity for ADM. These GPCRs include the ADM receptor L1 (ADMR-L1) [19] and the RDC1 receptor [20]; however, only CRLR coupled to RAMP2 has been firmly shown to be specific for ADM [16].

The aim of the current study was to determine whether ADM and a functional ADM receptor are present in the mouse retina. We immunocytochemically localized the expression of ADM and/or its receptors in the outer plexiform layer (OPL), inner plexiform layer (IPL), and in somata in the GCL, the INL, and the ONL. We confirmed the expression of *ADM*, *CRLR*, *RAMP2*, and *RAMP3* in the retina using reverse-transcriptase polymerase chain reaction (RT–PCR), and the presence of a functional ADM receptor by stimulating with ADM and showing an increase in cAMP-like immunoreactivity (LI), cyclic guanosine monophosphate (cGMP)-LI, and NO production. The results of our study indicate the existence of a functional ADM signaling cascade in neurons of the mouse retina.

## Methods

Unless specified otherwise, all reagents were purchased from Sigma (St. Louis, MO) or ThermoFisher Scientific (Waltham, MA). All images were labeled, arranged, and prepared for display using Corel Draw (Corel Corp., Ottawa, ON) unless otherwise noted.

### Animals

Adult, male C57BL/6 mice were purchased from Charles River Laboratories (Wilmington, MA) and were kept on 12 h:12 h light-dark cycles with free access to food and water. All animals were treated using protocols approved by the Boston University Charles River Campus Institutional Animal Care and Use Committee (IACUC).

### Immunocytochemistry

Animals were first heavily anesthetized using 3.0% IsoFlo isoflurane gas via a general anesthesia apparatus (Abbott Laboratories, North Chicago, IL) and then decapitated. The eyes were then enucleated and the anterior chambers immediately removed in ice-cold rodent balanced salt solution (BSS; 137 mM NaCl, 5 mM KCl, 2 mM CaCl_2_, 15 mM D-glucose, 1 mM MgSO_4_, 1 mM Na_2_HPO_4_, 10 mM HEPES, pH 7.4) and then placed directly into 4% paraformaldehyde in 0.1 M phosphate buffer (PB), pH 7.4 for 60 min. The eyecups were then cryoprotected in 30% sucrose in PB, embedded and frozen in optimal cutting temperature (OCT) embedding media (Tissue-Tek, Miles, Inc., Elkhard, IN) cut into 14-µm-thick cross sections using a cryostat, and then collected on SuperFrost/Plus slides (ThermoFisher Scientific). Immunochemistry (ICC) was performed using previously described conventional methods [21]. Sections on slides were washed with 0.1 M PB to remove excess OCT. All antiserum solutions were diluted in 0.1 M PB with 0.3% Triton X-100 (PBtx). Non-specific labeling was blocked by incubation in 5% normal goat, donkey, or rabbit serum (Jackson ImmunoResearch Labs, West Grove, PA) as appropriate, in PBtx for 1 h. Cross sections were incubated in primary antiserum overnight (Table 1). Following the incubation in primary antiserum, the sections were washed in PB and incubated for 2 h in fluorescent secondary antisera as appropriate. The secondary antisera used were an Alexa Fluor 488-conjugated donkey antigoat, donkey antirabbit, or donkey antimouse immunoglobulin G (IgG; Invitrogen, Grand Island, New York) and a CY3-conjugated donkey antigoat IgG (Jackson ImmunoResearch Laboratories, Inc. West Grove, PA) used at a dilution of 1:500 in PBTX. Incubation with only secondary antiserum was used as a control for non-specific secondary antiserum staining. To control for specificity, the ADM antisera (sc-16496) was incubated overnight with the synthetic peptide the antisera was raised against (ADM C-20 P sc-16496) before it was incubated with the cross sections. For double-labeling experiments, each secondary antiserum was excited by a separate laser (488 nm or 568 nm) and recorded in a separate channel. Images used for colocalization analysis were captured separately. The accuracy of the double labeling was confirmed by analyzing the labeling in single optical sections, while the specificity of the double labeling was confirmed by testing a given primary antisera with the opposite secondary antisera.

**Table 1:** List of antisera used

**Antigen**	**Antiserum**	**Source [catalog number],[reference number]**	**Working dilution**
ADM	Goat anti-ADM	Santa Cruz Biotechnology [sc-16496]	1:50
ADM	Rabbit anti-ADM	Santa Cruz Biotechnology [sc-33187]	1:100
CRLR	Rabbit anti-CRLR	GeneTex [GTX100340]	1:250
RAMP2	Rabbit anti-RAMP2	GeneTex [GTX108524]	1:250
Glutamine synthetase	Rabbit anti- glutamine synthetase	Sigma Aldrich [G2781] [45]	1:100
PKCα	Mouse anti- PKCα	Transduction Laboratories [610107] [45]	1:1,000
Calbindin	Mouse anti-calbindin-D28K	Sigma Aldrich [C94848] [45]	1:1,000
Calretinin	Rabbit anti-Calretinin	Millipore [MAB1568] [45]	1:1,000
nNOS	Rabbit anti-nNOS	Santa Cruz Biotechnology [sc-648] [22]	1:100
Glutamate	Rabbit anti-glutamate	Dr. David Pow [50]	1:100
GABA	Rabbit anti-GABA	Dr. David Pow [50]	1:100
Glycine	Rabbit anti-glycine	Dr. David Pow [50]	1:100
cAMP	Rabbit anti-cAMP	Calbiochem EMD Pharmaceuticals [116820] [51]	1:1,2500
cGMP	Rabbit anti-cGMP	Dr. Jan de Vente [52]	1:10,000

Finally, the slides were washed in PB and coverslipped with glycerol, and the fluorescent labeling was visualized using an Olympus Fluoview 300 confocal microscope (Olympus, Melville, NY). Image J image analysis software (Wayne Rasband, National Institute of Mental Health, Bethesda, MD) was used to convert images to inverted gray scale such that the immunoreactivity appeared black. The *z project* function of Image J was used to obtain a single image from a collapsed confocal optical stack.

### RNA isolation, reverse transcription, and reverse-transcriptase polymerase chain reaction

All animals were first anesthetized using isoflurane gas and then decapitated as described above. Following decapitation, the retinas were surgically isolated in RNase-free rodent BSS and placed immediately in 1.5 ml microcentrifuge tubes on dry ice. Six retinas (three animals) were typically pooled together to ensure an adequate amount of RNA. Total RNA was then isolated using a standard TRIzol reagent (Invitrogen, Carlsbad, CA) extraction followed by further purification using Qiagen’s RNeasy Kit (Qiagen, Valencia, CA) with modifications as described previously by Giove et al. [22]. Three hundred ﬁfty microliters of buffer RLT with β-mercaptoethanol was added per 100 µl of RNA. Two hundred ﬁfty microliters of 100% ethanol was then added, and the solution was mixed thoroughly before being placed into the provided RNeasy spin columns. The solution was centrifuged for 15 s at 10,000× g and the ﬂow-through discarded. The column was then washed twice with 500 µl of buffer RPE and centrifuged for an additional 1 min to remove residual liquid. The column was then transferred to a clean 1.5-ml centrifuge tube, and elution was performed per the manufacturer’s instructions. The RNA was then treated with rDNase (Ambion, Applied Biosystems) based on the manufacturer’s instructions to remove any DNA contaminants. The RNA was quantified using a NanoDrop spectrophotometer (ThermoFisher Scientific). cDNA was then made using the Verso cDNA Kit (ThermoFisher Scientific) and subsequently treated with 2 U of RNase H (ThermoFisher Scientific) at 37 °C for 20 min.

PCR was performed to amplify mouse *CRLR*, *RAMP2*, *RAMP3*, and *ADM* using the primers and conditions outlined in Uzan et al. [23] with 4 µl of cDNA converted from 1 µg of retinal RNA. The cDNA was amplified with Taq DNA polymerase and specific primers as indicated in Table 2. Ten µl of each reaction was then run on an ethidium bromide stained 1.2% agarose gel and imaged on Gel Doc XR (Bio-Rad, Hercules, CA) with its associated Quantity One software (Bio-Rad). Gel images were inverted for display. PCR products were confirmed by using automated DNA sequencing (SeqWright, Houston, TX).

**Table 2:** Primer Sequences Used.

***Gene***	**Primer Sequences (5′-3′)**	**PCR product (bp)**	**Annealing temp (°C)**
*ADM*	F: CGAAAGAAGTGGAATAAGTGGG	200	60
	R: GTTCATGCTCTGGCGGTAGCG		
*CRLR*	F: GCTGAATGACGTTGCAGC	480	60
	R: GCCTTCACAGAGCATCCA		
*RAMP2*	F: CATGGACTCTGTCAAGGACTG	153	55
	R: GTGTATCAGGTGAGCCT		
*RAMP3*	F: ACCTGTCGGAGTTCATCG	262	55
	R: ATCAGTGTGCTTGCTGCG		

Primer sequences used in this study for RT–PCR (ADM, CRLR, RAMP2, RAMP3).

### In vitro stimulation with adrenomedullin

Following the dissections described above, the isolated retinas were allowed to recover for 45 min at room temperature in Ames culture media. Isolated retinas were then preincubated for 20 min at room temperature in aerated BSS with the nonspecific phosphodiesterase (PDE) inhibitor 3-isobutyl-1-methylxanthine (1 mM). Following the preincubation with inhibitor 3-isobutyl-1-methylxanthine, the full-length rat ADM peptide (200 nM; American Peptide, Sunnyvale, CA) was added to the Ames media. This concentration was chosen based on an electrophysiological study of the effects of bath-applied ADM on rat brain slices that indicated this concentration produced clear electrophysiological effects [10]. After 1 h of stimulation at 37 °C with 95%O_2_/5%CO_2_, the isolated retinas were fixed for 2 h and then prepared for immunocytochemical analysis as described previously.

### Western blot analysis

Retinas from enucleated eyecups were removed and homogenized with ultrasonification in lysis buffer (50 mM Tris-HCl pH 7.5, 250 mM sucrose, 1 mM EDTA, 1% NP-40, 0.1% sodium dodecyl sulfate, 0.5% deoxycholate acid, 10 mM L-phenylmethylsulfonyl fluoride, 1% protease and phosphatase inhibitor cocktail [Halt; Thermo Scientific, Rockford, IL]). Retinal homogenates were spun down at 500× g for 5 min, and the resultant supernatants were used for analysis. Protein concentrations were determined with a modified Bradford assay. Forty micrograms of protein per lane were electrophoresed on 4%–20% Tris-glycine Pierce Protein precast gels (Thermo Scientific, Rockford, IL) at 150 V for 90 min and then were transferred for 2 h (80 V) to a polyvinylidene fluoride (PVDF) membrane (Millipore Corporation, Billerica, MA). Following washes in Tris-buffered saline with Tween-20 (TBST: 50 mM Tris, 150 mM sodium chloride, 1 mM magnesium chloride, and 0.25% Tween-20, pH 7.4), the membranes were blocked with 5% dried milk in TBST for 1.5 h at room temperature and incubated in primary antiserum diluted in 3% milk in TBST for 2 h. After the membranes were washed in TBST, an antirabbit IgG secondary antiserum conjugated to CY5 (1:1,000; Jackson ImmunoResearch) was applied for 1 h. After the membranes were washed several times in TBST, fluorescence was detected using a Typhoon Variable Mode Imager (GE Healthcare, Piscataway, NJ). Molecular weight determinations were done using Multi-Analyst (Bio-Rad) software.

### Nitrite analysis

Isolated mouse retinas were first allowed to recover for 30 min in Ames media. They were then incubated for 30 min with or without 1 mM of the nitric oxide synthase (NOS) inhibitor L-nitro-arginine methyl ester (L-NAME), followed by stimulation with ADM (200 nM; American Peptide) for 30 min. The use of the NOS inhibitor allowed us to confirm that the increased nitrite was due to the synthesis of NO and its decomposition to nitrite. The culture medium was then collected and used for nitrite analysis, and the retina was homogenized to determine total protein levels using a modified Bradford assay. Nitrite analysis was done using a modified Griess reaction using vanadium(III) chloride to convert nitrate to nitrite [24]. Nitrite levels were normalized to total protein levels, and the results were analyzed using a two-way ANOVA (n=6).

## Results

### Adrenomedullin was expressed in the mouse retina

**Figure 1:**
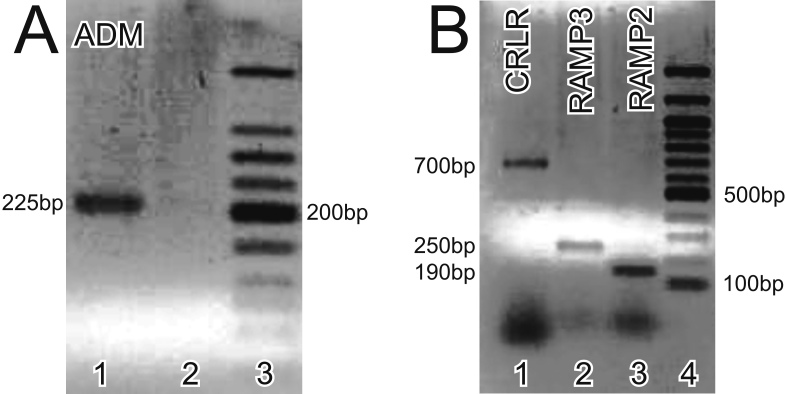
Adrenomedullin (ADM), calcitonin receptor like receptor (CRLR), and eceptor activity modifying protein (RAMPs) needed for ADM signaling are expressed in the retina. **A**: Reverse transcriptase (RT)–PCR using ADM specific primers [23] showed a single band at the expected size of approximately 225 bp. Lane 1=ADM RT–PCR; Lane 2=no template control; Lane 3=NEB 50 bp ladder (New England BioLabs, Ipswich, MA), darkest band=200 bp. **B**: RT–PCR using primers described by Uzan et al. [23] confirmed the expression of CRLR (Lane 1, approximately 700 bp), RAMP3 (Lane 2, approximately 250 bp), and RAMP2 (Lane 3, approximately 190 bp). Lane 4=NEB 100 bp ladder.

**Figure 2:**
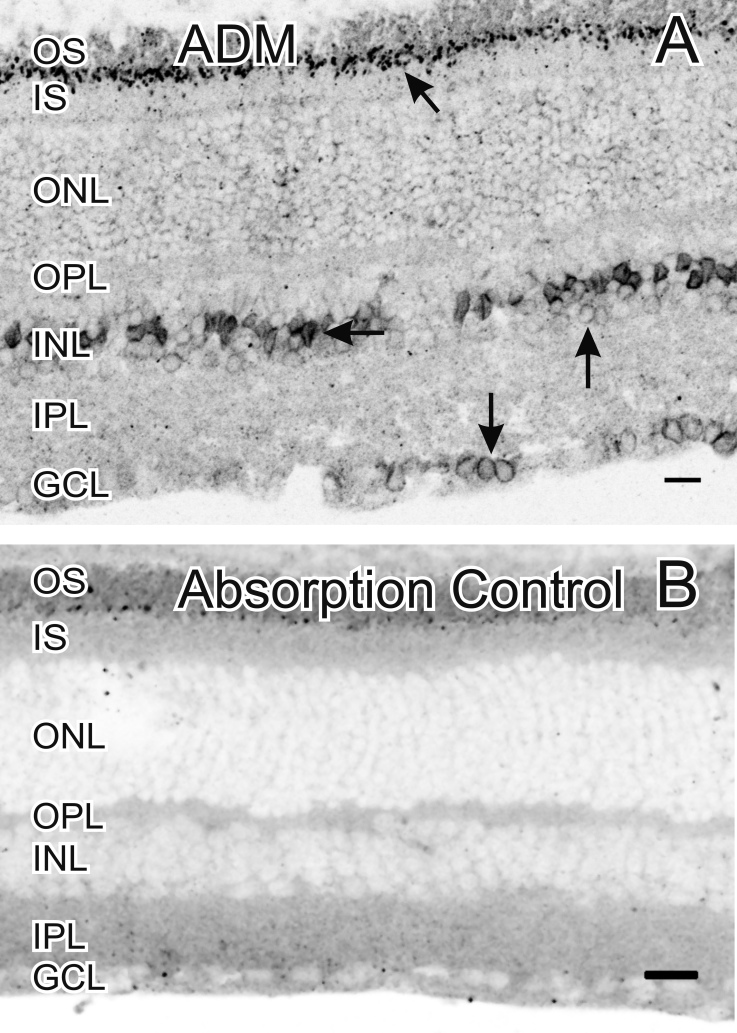
Adrenomedullin (ADM) in the mouse retina. **A**: ADM-like immunoreactivity (LI) was predominantly localized in cell somata in the middle layer of the inner nuclear layer (INL; horizontal arrow), and in presumptive ganglion cell somata in the ganglion cell layer (GCL; down arrow), and near the outer segments (OS; diagonal arrow). We also observed faint ADM-LI in the cytoplasm of somata in the INL adjacent to the inner plexiform layer (IPL; up vertical arrow). **B**: There was no labeling in control sections in which the ADM primary antiserum was preincubated with the peptide the antiserum was raised against. Inner segments=IS, Scale bars=20 μm.

We confirmed the expression of *ADM* in the retina using RT–PCR (Figure 1A). ADM-LI was detected weakly throughout the retina but was strongly localized near the photoreceptor outer segments, in elongated somata in the middle of the INL, and in some large somata in the GCL (Figure 2A) compared to an absorption control (Figure 2B) and a no primary control (not shown). We also observed faint ADM-LI in the cytoplasm of somata in the INL adjacent to the IPL.

Although we did not confirm the specificity of either of the ADM antisera using western blots, we established the immunocytochemical specificity of the antisera because each ADM antiserum was directed against a different epitope on ADM. The one antiserum from Santa Cruz (Santa Cruz, CA, sc-16496) was directed against an epitope mapping within an internal region of ADM, while the other antiserum (sc-33187) was directed against an epitope corresponding to amino acids 1–185 representing full-length ADM of human origin. Although both antisera showed qualitatively similar labeling that indicated the specificity of the labeling, we show only the labeling with the sc-16496 antiserum as it was more robust. Additionally, we show no staining in control tissue when ADM was preabsorbed with the synthetic peptide (Figure 2B). Using an *ADM* knockout mouse to test for AM antibody specificity was not possible as homozygous AM (AM^−/−^) knockout mice die in utero around mid-gestation from systemic hemorrhage and edema resulting from the fragility of their vasculature [25].

Although there was some faint ADM-LI in the outer nuclear layer, it does not seem likely that the strong punctate ADM-LI near the photoreceptor outer segments is actually in the outer segments themselves. Given that ADM has been previously localized in the RPE [8,12], it is more probable that the punctate ADM-LI is either from ADM secreted by the RPE, from broken RPE cell fragments sticking to the photoreceptor outer segments, or from RPE processes interdigitating with the outer segments.

**Figure 3:**
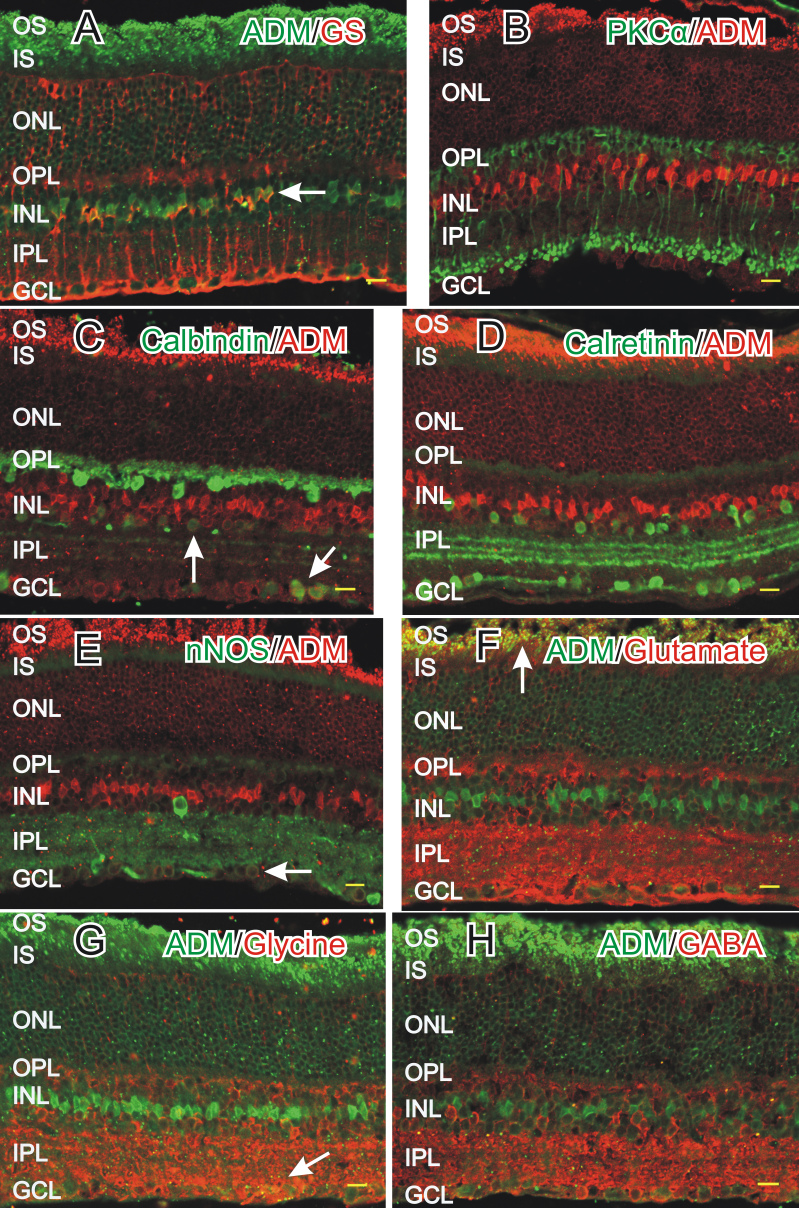
Colocalization of adrenomedullin (ADM) with cell specific markers was done to accurately identify cell types with ADM-like immunoreactivity (LI). In all figures, ADM-LI was localized near the photoreceptor outer segments and in somata in the inner nuclear layer (INL) and the ganglion cell layer (GCL). **A**: Glutamine synthetase (GS, red) labeled Müller cells, and colocalized with ADM (green) in their somata in the INL (horizontal arrow). **B**: Protein kinase C α-like immunoreactivity (PKCα-LI; green) was present in rod bipolar cells and did not colocalize with ADM-LI (red). **C**: Calbindin-LI (green) was localized in horizontal cells in the INL and their process in the OPL, as well as in somata in the INL and GCL. ADM-LI (red) colocalized with calbindin-LI in somata in the lower tier of the INL that borders the IPL (vertical arrow) and in somata in the GCL (diagonal arrow). **D**: Calretinin-LI (green) was localized in amacrine cell somata in the INL, in processes in the inner plexiform layer (IPL), and within somata in the GCL. ADM-LI (red) did not colocalize with calretinin-LI. **E:** neuronal nitric oxide synthase (nNOS)-LI (green) was localized in select amacrine cell somata in the INL, in processes in the IPL, and in somata in the GCL. Some somata with nNOS-LI in the GCL colocalized with ADM-LI (red) (arrow). **F:** Glutamate-LI (red) was localized near the photoreceptor outer segments, in the OPL, in somata in the INL and the GCL, and as diffuse staining in the IPL. ADM-LI (green) colocalized with glutamate near the outer segments (arrow). **G:** Glycine-LI (red) was localized in the INL, in the OPL and the IPL, and in somata in the INL and the GCL. Glycine-LI was not colocalized with ADM-LI (green) in any somata, but there was some potential colocalization in puncta in the IPL (arrow). **H:** gamma-aminobutric acid (GABA)-LI (red) was localized to somata in the ONL, processes in the OPL, somata in the INL, diffusely in the IPL, and somata in the GCL. ADM-LI (green) did not colocalize with GABA-LI. Scale bars=20 µm.

To accurately identify the specific cell types that were positive for ADM-LI, cross sections were double-labeled with ADM antiserum and cell specific markers. Colocalization of ADM-LI with the Müller cell marker glutamine synthetase (Figure 3A) demonstrated that the ADM-LI positive somata in the middle of the INL were Müller cells. The other cell somata in the middle of the INL were not rod bipolar cells as there was no colocalization of ADM-LI with the rod bipolar cell marker protein kinase Cα (Figure 3B). Additionally, ADM-LI was not localized in horizontal cells as demonstrated by the horizontal cell marker calbindin (Figure 3C). Although ADM-LI was not in horizontal cells, it was colocalized with calbindin-LI in somata in the INL on the border with the IPL, and within somata in the GCL, supporting that ADM-LI is in amacrine cells and either displaced amacrine or ganglion cells. ADM-LI positive cells did not colocalize with amacrine cells that were positive for calretinin-LI (Figure 3D) or neuronal nitric oxide synthase (nNOS)-LI (Figure 3E). However, some somata with nNOS-LI in the GCL did colocalize with ADM-LI. ADM-LI was colocalized with the excitatory neurotransmitter glutamate in the photoreceptor outer segments (Figure 3F), but not within the rest of the retina. ADM-LI did not colocalize with the inhibitory neurotransmitters glycine (Figure 3G) or gamma-aminobutyric acid (Figure 3H) in amacrine cell somata, but there was some potential colocalization of glycine in occasional puncta in the IPL.

### Adrenomedullin receptors were present in the mouse retina

**Figure 4:**
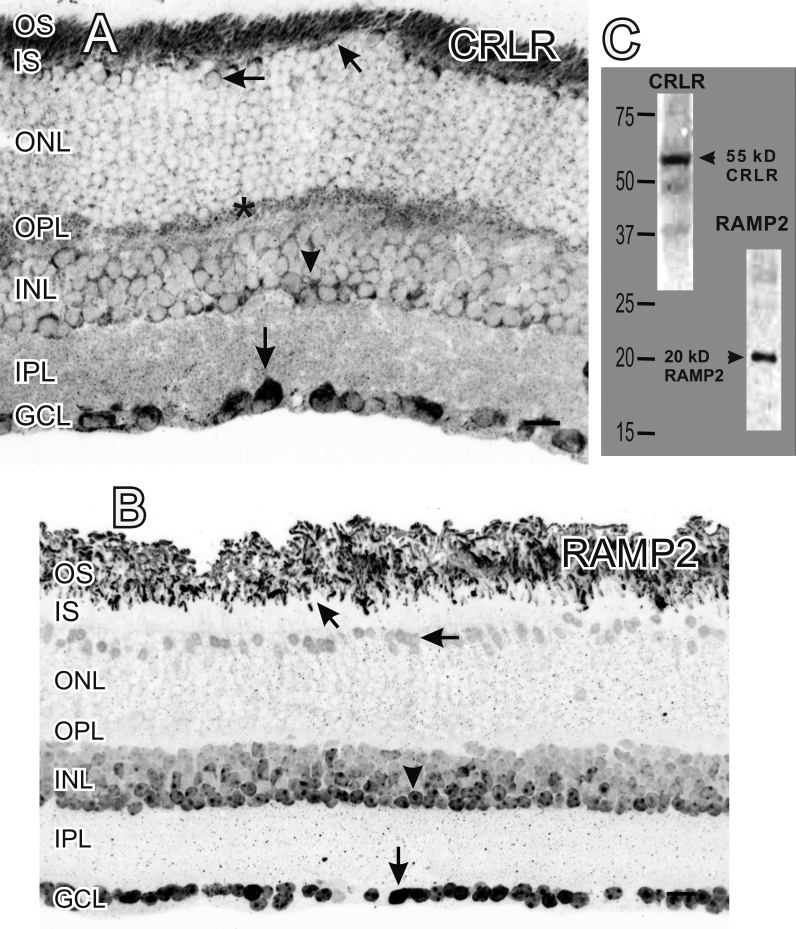
Immunocytochemical localization of the adrenomedullin (ADM) receptor calcitonin receptor like receptor (CRLR) and receptor activity modifying protein (RAMP2). **A:** The ADM receptor CRLR-like immunoreactivity (LI) was localized near the outer segments of photoreceptors (diagonal arrow), in faint somata in the outer nuclear layer (ONL; horizontal arrow), in puncta in the outer plexiform layer (OPL; asterisk), in select cell somata in the inner nuclear layer (INL; arrowhead), in delicate puncta in the inner plexiform layer (IPL), and in numerous somata in the ganglion cell layer (GCL; vertical arrow). **B**: RAMP2-LI was localized near the photoreceptor outer segments (diagonal arrow), in somata in the ONL (horizontal arrow), and in somata in the INL (arrowhead) and GCL (vertical arrow). Scale bars=20 µm. **C**: western blot of mouse retinal homogenate probed with the same CRLR and RAMP2 antisera used for immunocytochemistry. Both antisera recognized single proteins with the correct molecular weights.

**Figure 5:**
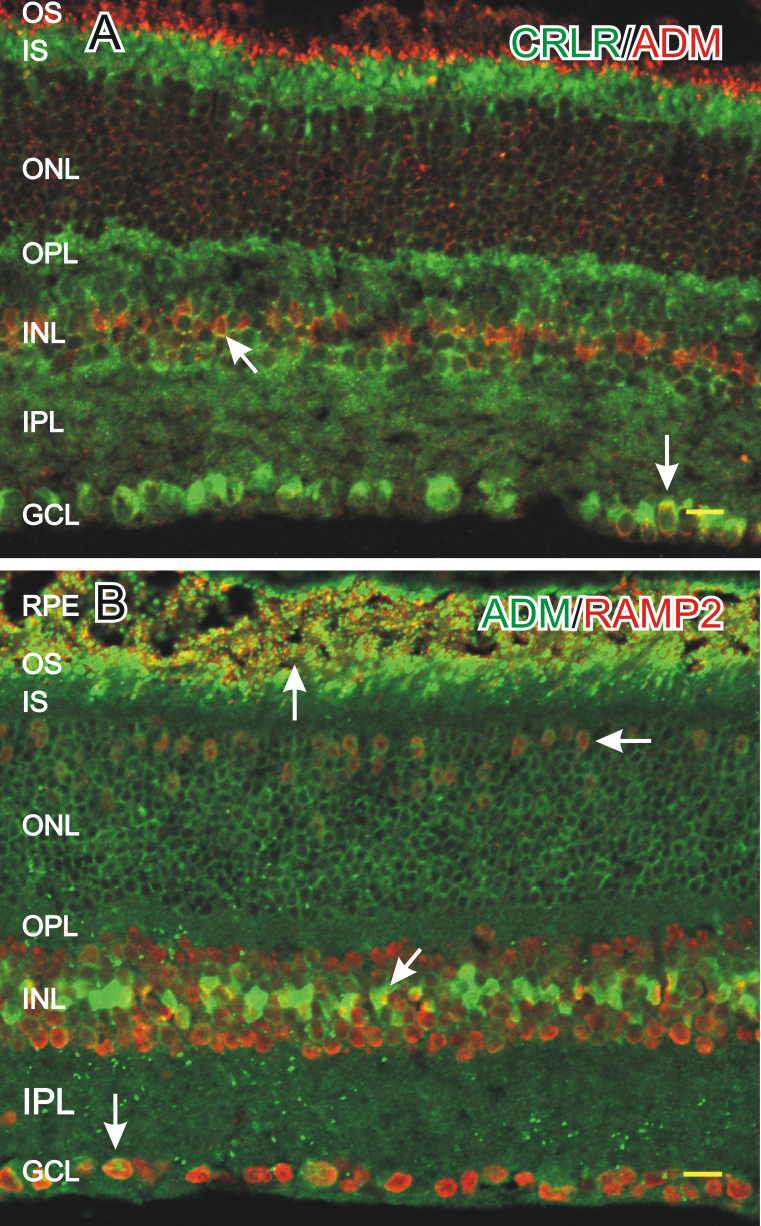
Colocalization of adrenomedullin (ADM) with its receptor. **A**: ADM-like immunoreactivity (LI; red) was colocalized with calcitonin receptor like receptor (CRLR; green) in somata in the inner nuclear layer (INL; diagonal arrow) and somata in the ganglion cell layer (GCL; down arrow). **B**: ADM-LI (green) was colocalized with RAMP2 near the outer segments (up arrow), in cell somata in the ONL (horizontal arrow), and in somata in the INL (diagonal arrow) and the GCL (down arrow). Scale bars=20 µm.

We used RT–PCR to determine whether the primary ADM receptor, *CRLR*, and its associated receptor activity modifying proteins were expressed in the retina. We detected the expression of *CRLR*, *RAMP2*, and *RAMP3* (Figure 1B). We then used immunocytochemistry to localize the ADM receptors in the retina. Because we were unable to find a reliable antiserum against RAMP3, we focused on CRLR and RAMP2. CRLR-LI was detected primarily in large somata in the GCL, in faint puncta in the OPL, in photoreceptor outer segments or the RPE, in photoreceptor inner segments, and in the cytoplasm of numerous faint somata in the ONL and the INL (Figures 4A and Figure 5A). We found RAMP2-LI was strongly localized in many somata in the INL and the GCL, in faint somata in the outer ONL, and in photoreceptor outer segments (Figure 4B). The RAMP2-LI in the INL was present in numerous somata that resembled amacrine cells by location and morphology. The specificity of the CRLR and RAMP2 antisera was confirmed using western blots that recognized proteins of the correct molecular weight (Figure 4C). Labeling in CRLR or RAMP2 knockout mice as a control for specificity was not possible as *CRLR* (*CRLR*^−/−^) and *RAMP2* (*RAMP2*^−/−^) knockout mice are embryonic lethal due to vascular complications [26,27].

To determine if ADM-LI and its receptors were colocalized, cross sections were double-labeled with ADM antisera and either CRLR or RAMP2 antisera. ADM-LI was colocalized with CRLR in somata in the INL and the GCL but was not colocalized in the outer segments or the OPL (Figure 5A). ADM-LI was colocalized with RAMP2 in the outer segments, in somata in the ONL, as well as in some somata in the INL and the GCL (Figure 5B).

### Adrenomedullin receptors were functional in the mouse retina

#### Adrenomedullin simulates production of cyclic adenosine monophosphate and cyclic guanosine monophosphate

**Figure 6:**
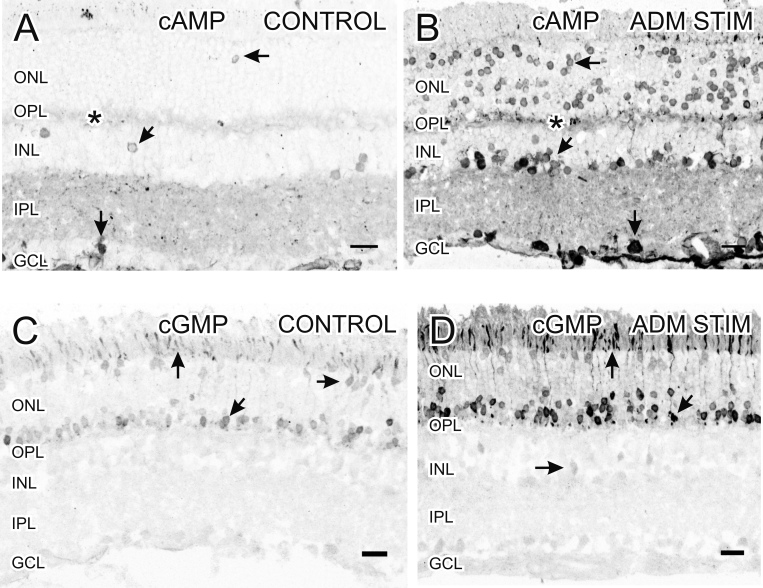
Stimulation with 200 nM adrenomedullin (ADM) increases cyclic adenosine monophosphate (cAMP)-like immunoreactivity (LI) and cyclic guanosine monophosphate (cGMP)-LI. **A**: In the control retinas, cAMP-LI was in isolated somata in the outer nuclear layer (ONL; horizontal arrow), in faint staining in the outer plexiform layer (OPL; asterisk), in sparse somata in the inner nuclear layer (INL; diagonal arrow), in diffuse puncta in the inner plexiform layer (IPL), and in somata in the ganglion cell layer (GCL; down arrow). **B**: In retinas stimulated with ADM, there was an overall increase in cAMP-LI, with increased numbers of photoreceptor somata (horizontal arrow), puncta in the OPL (asterisk), and somata in the INL (diagonal arrow) and the GCL (down arrow). **C**: In the control retinas, faint cGMP-LI was in some photoreceptor outer segments (vertical arrow) and in somata in the inner (diagonal arrow) and outer (horizontal arrow) ONL. **D**: In retinas stimulated with ADM, there was an increase in the number and intensity of labeled photoreceptor outer segments (up arrow) and somata in the inner region of the ONL (diagonal arrow). There were also some faint somata in the INL (horizontal arrow). Scale bars=20 µm.

Isolated retinas were stimulated with ADM (200 nM), and immunocytochemistry was then used to detect the downstream second messengers cAMP and cGMP (Figure 6). We found an increase in cAMP-LI and cGMP-LI when isolated retinas were stimulated with ADM.

In the control retinas, faint cAMP-LI was localized in rare somata in the ONL, faint labeling in the OPL, sparse somata in the INL, isolated puncta in the IPL, and in some faint somata in the GCL (Figure 6A). There was an overall increase in cAMP-LI when retinas were stimulated with ADM. We found a large increase in the number of photoreceptor somata, increased staining in the OPL and the IPL, and many more strongly labeled somata in the INL and the GCL (Figure 6B). Increased cAMP-LI in axons in the ganglion cell axon layer supported that at least some of the labeled somata in the GCL were ganglion cells.

Faint cGMP-LI was localized in photoreceptor outer segments and in somata in the outer retina in the control retinas (Figure 6C). There was a dramatic increase in the number and intensity of labeled photoreceptor outer segments and somata in retinas stimulated with ADM (Figure 6D). There were also some faint somata in the INL.

#### Adrenomedullin stimulates an increase in nitric oxide degradation products

**Figure 7:**
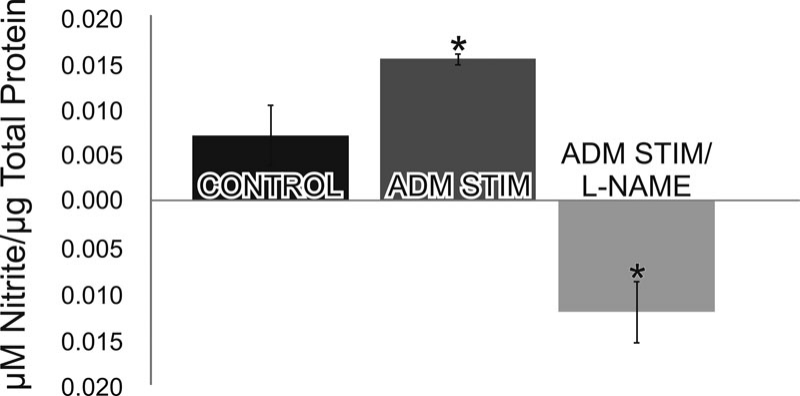
Stimulation with adrenomedullin (ADM) increases nitric oxide (NO) production. In isolated retinas stimulated with ADM (200 nM), there was a statistically significant increase in nitrite (i.e., NO) production. There was a statistically significant decrease in nitrite production when the isolated retinas were stimulated with ADM in the presence of the nNOS inhibitor Nω-Nitro-L-arginine methyl ester (L-NAME). Asterisks denote p<0.001 (two way ANOVA n=6; error bars represent the standard deviation).

Nitrite analysis was used as a measure of NO production in isolated retinas stimulated with ADM. There was a statistically significant increase (p<0.001) in NO production in retinas that were stimulated with ADM (200 nM), and a statistically significant decrease (p<0.001) in NO production when the isolated retinas were stimulated with ADM in the presence of the NOS inhibitor L-NAME (1 mM; Figure 7). The decrease in nitrite to below control levels seen with L-NAME indicated that basal levels of NOS activity were inhibited as well.

## Discussion

### Presence of a functional adrenomedullin signaling pathway in the retina

Although ADM was first described as a vasodilator [1], it has become clear that ADM has many other diverse functions, including neurotransmission [4,28]. Our anatomic localization confirms and adds detail to the previous localization of ADM in the mouse retina [13]. We detected ADM in several neural layers of the mouse retina, with the strongest expression in Müller cell somata in the INL and displaced amacrine and/or ganglion cell somata in the GCL, and more diffusely in amacrine cell somata in the INL. The localization of the ADM positive somata in the middle of the INL and the close numerical correlation of the somata with ADM to the number of Müller cell processes strongly suggest that these somata are from Müller cells. Moreover, detailed anatomic studies of mouse bipolar cells indicate that most bipolar cell somata are located more closely to the outer plexiform layer and not in the middle of the INL [29]. However, some of the somata in the INL could be bipolar cells.

**Figure 8:**
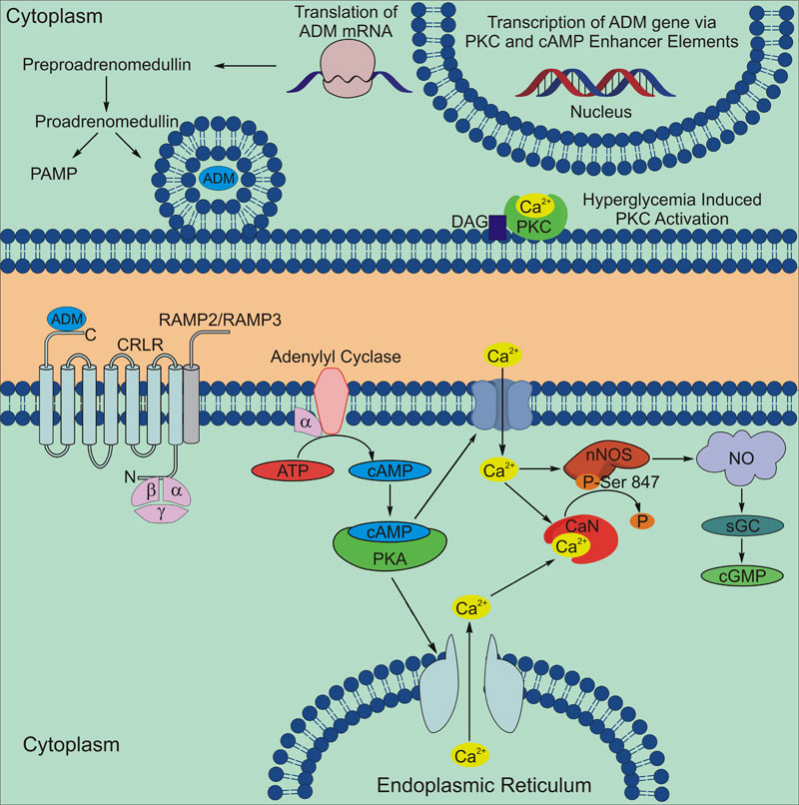
Summary diagram of the proposed adrenomedullin (ADM) signaling pathway in the retina. Protein kinase C (PKC) activation can lead to increased transcription of the ADM gene via a PKC enhancer element. The ADM precursor preproadrenomedullin is cleaved to proadrenomedullin, which is then cleaved into the secreted peptide ADM and the proadrenomedullin NH_2_-terminal peptide (PAMP). ADM is secreted and binds to the G-protein coupled receptor calcitonin receptor like receptor (CRLR) that is associated with either the receptor activity modifying protein RAMP2 or RAMP3 to activate a signaling cascade that increases cyclic adenosine monophosphate (cAMP) by activating adenylyl cyclase. Increases in cAMP levels activate protein kinase A (PKA), which increases calcium levels by opening membrane calcium channels or by releasing intracellular calcium stores. The overall increase in intracellular calcium can increase nitric oxide (NO) production by directly stimulating nNOS or by activating nNOS through the activation of the calcium-activated phosphatase, calcineurin (CaN), which dephosphorylates nNOS at an inhibitory phosphorylation site at serine^847^. Increases in NO production can then increase in cGMP synthesis by activating soluble guanylyl cyclase (sGC).

Most importantly, we found that the primary receptor for ADM, CRLR, and its associated RAMP2 had largely overlapping expression with ADM, especially in Müller cell somata and in presumptive ganglion cell somata in the GCL. The increased levels of cAMP-LI, cGMP-LI, and nitrite in response to stimulation with the ADM peptide provided further evidence of a functional ADM receptor and an ADM/NO signaling pathway in the mouse retina (Figure 8).

ADM production can be regulated in several ways. The ADM gene has been shown to have cAMP and protein kinase C enhancer elements [30], and protein kinase C and cAMP are involved in many aspects of retinal function. In this report, we provide evidence that ADM stimulation can increase production of the NO degradation product nitrite. We have previously shown that every cell type in the retina can make NO [31]. Interestingly, NO itself has been shown to stimulate adrenomedullin secretion and gene expression [32]. Therefore, the production and secretion of ADM in the retina can theoretically be regulated by every cell type in the retina.

### Adrenomedullin receptors in the retina

ADM is a secreted peptide that can function in an autocrine or paracrine manner, as seen in rodent development [33]. In addition, ADM normally circulates in the bloodstream [34], and ADM can cross the blood–brain barrier (BBB) [35]. Since the BBB and the blood–retinal barrier (BRB) are similar, this raises the possibility that the retina can respond to circulating levels of ADM in addition to locally secreted ADM. Conversely, ADM’s ability to cross the BRB may account for the increased plasma levels of ADM seen in patients with retinitis pigmentosa [9].

**Figure 9:**
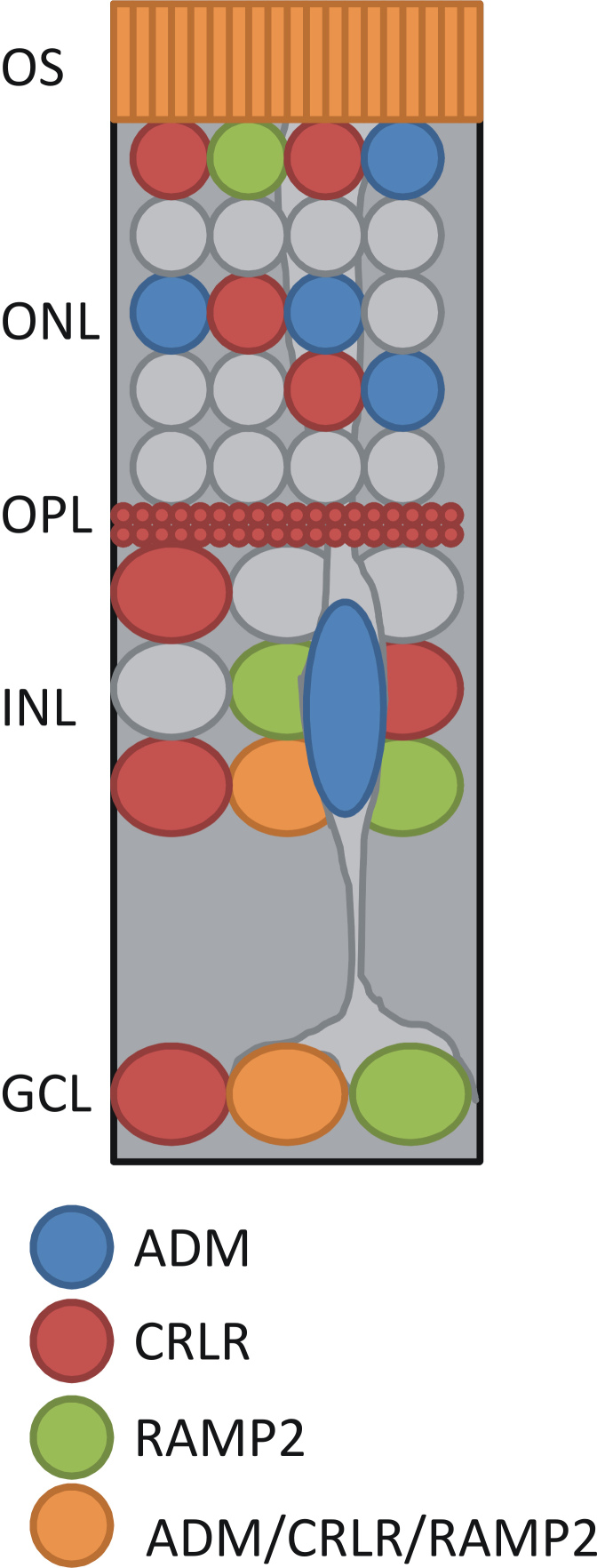
Summary of the cellular localization of the ADM signaling pathway in the retina. ADM was present near the outer segments (OS), in somata in the INL and the GCL, and in Müller cells. CRLR and RAMP2 were present near the OS, and in somata in the ONL, INL, and GCL. ADM, CRLR, and RAMP2 were colocalized near the OS, and in somata in the INL and the GCL.

Although the CRLR/RAMP2 receptor complex would clearly be functional when it is inserted into the cell membrane, there can also be unique intracellular punctate localization of RAMP2 (Figure 9). The binding of agonists often leads to the rapid internalization of many cell surface G-protein coupled receptors, including adrenomedullin binding to CRLR/RAMP2 receptors [18]. This internalized receptor is then incorporated into endosomes and then degraded. The punctate labeling we see might represent RAMP2 within endosomes.

The strong expression of ADM, CRLR, and increased cAMP-LI in response to ADM in somata in the GCL cells suggests that ADM functions in an autocrine manner in the retina as well. However, the localization of ADM-stimulated increases in cAMP-LI and cGMP-LI in the ONL and cGMP-LI in photoreceptor outer segments suggests that ADM can also function in a paracrine fashion in the retina. Given our localization of CRLR-LI and RAMP2-LI in the outer retina, ADM coming from the pigment epithelium [8] could influence these receptors in the outer retina in a paracrine fashion.

Additional potential ADM receptors, ADMR-L1 [19], RDC1 [20], and hrhAM [36], were not investigated in our study. However, the results of studies examining the affinity of ADM for these other receptors are inconsistent, suggesting that the only truly specific ADM receptor is CRLR [16] when it is associated with RAMP2. In fact, CRLR has complete specificity for ADM only when CRLR is associated with RAMP2, because when CRLR is associated with RAMP3, CRLR can bind to either ADM or the related peptide amylin [37]. Although there are no published studies localizing amylin in the retina, like ADM, amylin can cross the BBB [38], suggesting that amylin could still exert an effect on the retina if the CRLR/RAMP3 combination is present and activated.

### Adrenomedullin activation of downstream signaling

Although ADM has been implicated in several ocular diseases, it is important to consider what role ADM may play in normal retinal function. Many studies have focused on the diverse roles of NO in the function of retinal neurons [39]. NO can have many neurochemical effects on retinal neurons, such as modulating gamma-aminobutyric acid and glycine release [40], influencing cholinergic signaling [41], and modulation of gap junctional conductance between horizontal cells [42] and between AII amacrine cells and cone bipolar cells [43]. Xu and Krukoff [11] have reported that ADM can increase NO production in cortical neurons through the activation of the calcium-activated phosphatase, calcineurin, which dephosphorylates neuronal NOS (nNOS) at an inhibitory phosphorylation site at serine^847^. Previous studies show that the expression of calcineurin [44] and nNOS [22,45] can overlap in the GCL, IPL, INL, and OPL, which is where we found ADM and its receptors. Therefore, the ADM signaling pathway might modulate NO production in normal and pathological retinas. For example, increases in retinal ADM may increase retinal NO through a dephosphorylation of nNOS as described by Xu and Krukoff [11].

The fact that CRLR and RAMP2 are located in many somata in the inner retina while stimulation with ADM primarily increases cAMP-LI and cGMP-LI in the outer retina indicates that activation of CRLR may also activate other signaling pathways in the inner retina. For instance, in the rat heart ADM mobilizes Ca^2+^ release from intracellular ryanodine- and thapsigargin-sensitive Ca^2+^ stores, increases Ca^2+^ influx through L-type Ca^2+^ channels, and activates protein kinase C [46]. In vascular endothelial cells, ADM can transactivate the tyrosine kinase domain of the vascular endothelial growth factor receptor 2 [47]. Finally, intracerebroventricular administration of ADM increases the expression of *c-fos* in neurons in rat cardiovascular-related brain nuclei [48]. The more extensive localization of RAMP2-LI compared to CRLR-LI may be a reflection of the association of RAMP-2 with other G-protein coupled receptors in addition to CRLR [49]. Future studies should analyze the presence of additional ADM receptors, such as RDC1, and ADMR-L1, although they are not optimal ADM receptors. It will also be important to determine whether there are changes in ADM expression or in the activity of its downstream signaling components in pathologies known to increase ADM levels such as diabetic retinopathy, glaucoma, retinitis pigmentosa, or uveitis. A better understanding of the activation or inhibition of the system will also provide insight into the role ADM plays in normal retinal synaptic processing. Given the widespread localization of ADM and its receptors in the retina, ADM and its signaling pathways may prove to be important mediators of retinal function and may provide valuable new targets for ameliorating retinal pathology.
